# Supplementary material for: Genetically Encoded and Modular SubCellular Organelle Probes (GEM-SCOPe) reveal lysosomal and mitochondrial dysfunction driven by PRKN knockout
Source: bioRxiv. 2025 Jun 2:2024.05.21.594886. Preprint. [Version 3] doi: 10.1101/2024.05.21.594886 (PMC11230217; doi:10.1101/2024.05.21.594886)
Supplement: Supplement 2 — Details for all plasmids, including Addgene catalog #, subcellular localization, fluorophore, antibiotic resistance, and in which figure each plasmid was used. [file NIHPP2024.05.21.594886v3-supplement-2.pdf]

bioRxiv preprint doi: https://doi.org/10.1101/2024.05.21.594886; this version posted June 2, 2025. The copyright holder for this preprint (which was not certified by peer review) is the author/funder, who has granted bioRxiv a license to display the preprint in perpetuity. It is made available under aCC-BY 4.0 International license.

Fig. S1: GEM-SCOPe supports a diversity of modifications and applications (A) Representative images of astrocytes expressing COX8A-Emerald under the control of a doxycycline inducible promoter. Astrocytes were treated with a vehicle (DMSO; top) or doxycycline (bottom) for 72 hours. Nuclei were stained with Hoechst33342. **(B)** Representative images of a mixed population of astrocytes with nuclei expressing different H2B-fusion fluorophores. Astrocytes were transduced with a single lentivirus and then plated together. **(C)** qPCR for endogenous *LAMP1* (left) and lentiviral *LAMP1* (right) on RNA from astrocytes transduced with a virus localized to the lysosome (LAMP1-mCherry) or a virus not localized to the lysosome (COX8A-Emerald). Endogenous *LAMP1* qPCR primers were designed to amplify a sequence of *LAMP1* that is not included in the lentivirus and lentiviral *LAMP1* qPCR primers were designed to amplify the signal sequence of *LAMP1* and the beginning of the mCherry sequence. Bars represent mean and error bars represent standard deviation (n = 3 independent transductions). Statistical differences were determined by unpaired t-test; (left) $p = 0.07$; (right) $p = 0.002$. **(D)** Schematic of the lentiviral construct used in the following panels localizing Lemon, a pH responsive fluorophore, to the mitochondria with the targeting sequence of COX8A. Lemon undergoes Forster Resonance Energy Transfer (FRET) under alkaline conditions. **(E)** Representative images and quantification of astrocytes transduced with COX8A-Lemon and treated with a vehicle (DMSO; top) or 2 μM oligomycin (bottom) for 24 hours. Images were acquired with a 455nm-excitation laser and a 515-excitation laser. Far right: ratiometric representation of the emission in the yellow channel divided by the emission in the cyan channel and then pseudo colored so that orange, yellow and white indicate more relative yellow emission (more alkaline) while black and purple represent more cyan emission (more acidic). Nuclei were stained with DRAQ5. Central bars represent mean and error bars represent standard deviation (n = 3; each replicate is an average 36 images). Scale bars = 50 μm. For all graphs, * $p< 0.05$, ** $p < 0.01$, *** $p < 0.001$, **** < 0.0001.

bioRxiv preprint doi: https://doi.org/10.1101/2024.05.21.594886; this version posted June 2, 2025. The copyright holder for this preprint (which was not certified by peer review) is the author/funder, who has granted bioRxiv a license to display the preprint in perpetuity. It is made available under aCC-BY 4.0 International license.

Fig. S2: Astrocyte proliferation is activated by FBS and inhibited by MG-132 (A) Representative images of astrocytes transduced with H2B-Emerald lentivirus after 0 hrs (top) or 48 hrs (bottom) of treatment with a vehicle (DMSO; left), 10% fetal bovine serum (FBS; middle), or MG132 (right). Scale bars = 50 µm. **(B)** Quantification of number of H2B-Emerald nuclei per image field 0 hours and 48 hours after treatment. Dots represent mean values and error bars represent standard deviation (n = 6 (n = 3 for MG-132 treatment) per time point; each replicate is an average over 25 images). Scale bars = 50 µm. All images were acquired on the CX7 HCS platform with a 20x objective lens. For all graphs, * $p< 0.05$, ** $p < 0.01$, *** $p < 0.001$, **** < 0.0001.

Fig. S3: *PRKN* knockout and overexpression (A) Sequencing alignment of *PRKN*$^{-/-}$ astrocytes with the *PRKN* coding sequence showing a 62bp deletion in exon 3 **(B)** qPCR for *PRKN* in *PRKN*$^{+/+}$, *PRKN*$^{-/-}$, and *PRKN*$^{-/-}$ with *PRKN*-overexpression. qPCR primers were designed to amplify the region deleted in the *PRKN*$^{-/-}$ cells. Bars represent mean and error bars represent standard deviation (1-way ANOVA with Tukey's HSD; *PRKN*$^{+/+}$ vs *PRKN* knock-in: $p = 0.0004$; *PRKN*$^{-/-}$ vs *PRKN* knock-in: $p = 0.0004$; n = 3 independent transductions). For all graphs, * $p< 0.05$, ** $p < 0.01$, *** $p < 0.001$, **** < 0.0001.

Fig. S4: Mitochondrial fragmentation exhibits a dose dependent response to oligomycin-induced stress (A) Representative images of astrocytes transduced with COX8A-Emerald and treated with a vehicle (DMSO), 1µM, 2 µM, or 5 µM oligomycin for 4 hours. Nuclei were stained with Hoechst33342. Scale bars = 50 µm **(B)** Quantification of mitochondrial features indicative of mitochondrial network fragmentation: mean area, perimeter, aspect ratio, number of branches, branch length, and number of branch endpoints. Central bars represent mean and error bars represent standard deviation (1-Way ANOVAs with Dunnett's test; n = 3; each replicate is an average over 36 images). For all graphs, * $p< 0.05$, ** $p < 0.01$, *** $p < 0.001$, **** < 0.0001.

bioRxiv preprint doi: https://doi.org/10.1101/2024.05.21.594886; this version posted June 2, 2025. The copyright holder for this preprint (which was not certified by peer review) is the author/funder, who has granted bioRxiv a license to display the preprint in perpetuity. It is made available under aCC-BY 4.0 International license.

Figure S1

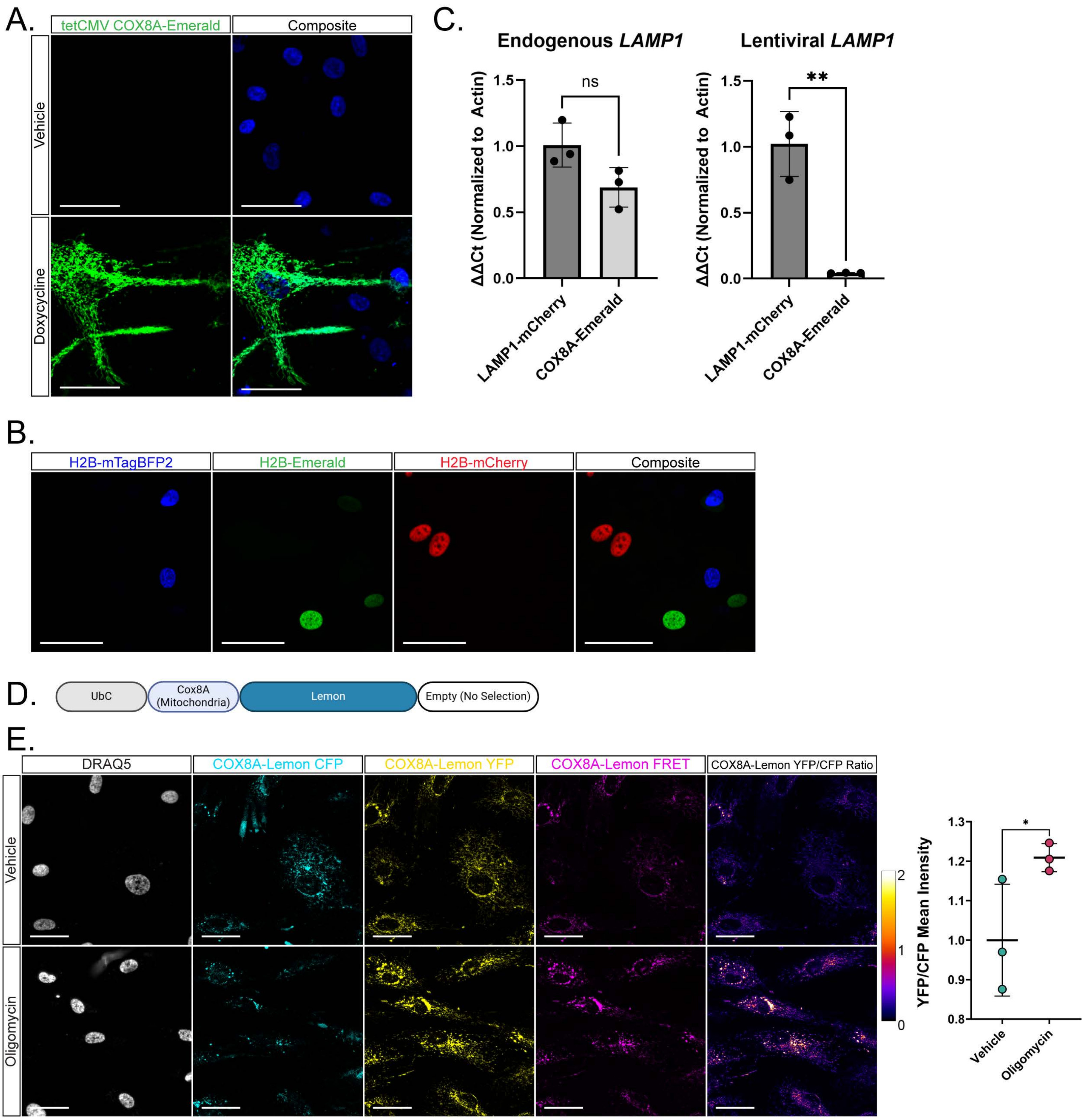

bioRxiv preprint doi: https://doi.org/10.1101/2024.05.21.594886; this version posted June 2, 2025. The copyright holder for this preprint (which was not certified by peer review) is the author/funder, who has granted bioRxiv a license to display the preprint in perpetuity. It is made available under aCC-BY 4.0 International license.

Figure S2

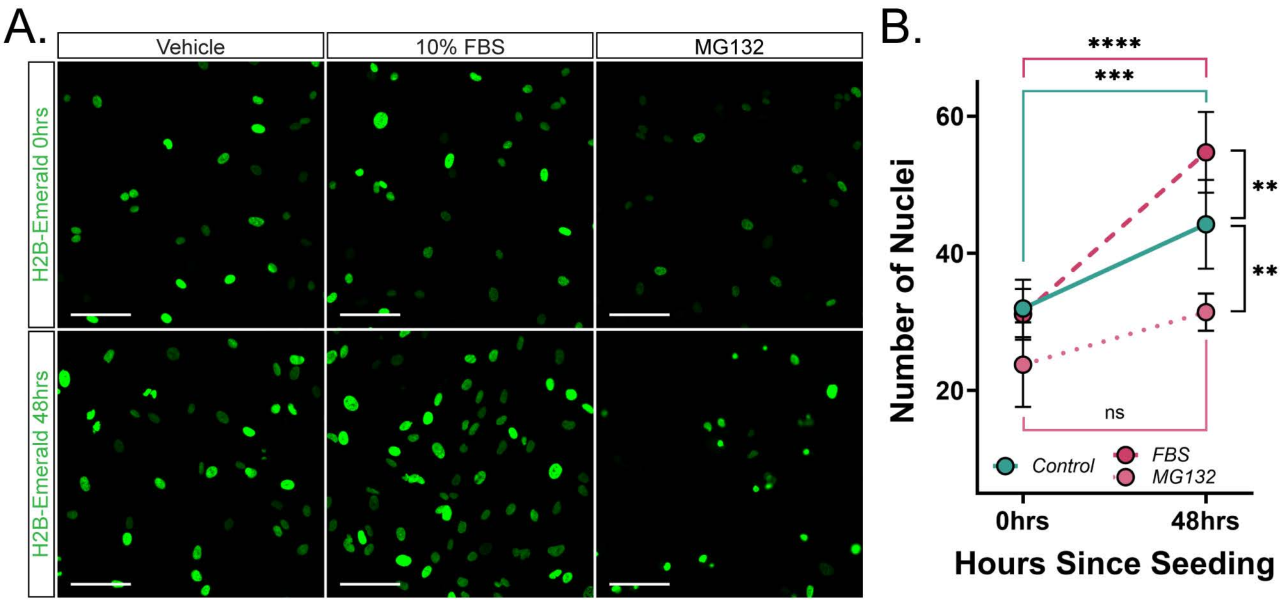

bioRxiv preprint doi: https://doi.org/10.1101/2024.05.21.594886; this version posted June 2, 2025. The copyright holder for this preprint (which was not certified by peer review) is the author/funder, who has granted bioRxiv a license to display the preprint in perpetuity. It is made available under aCC-BY 4.0 International license.

Figure S3

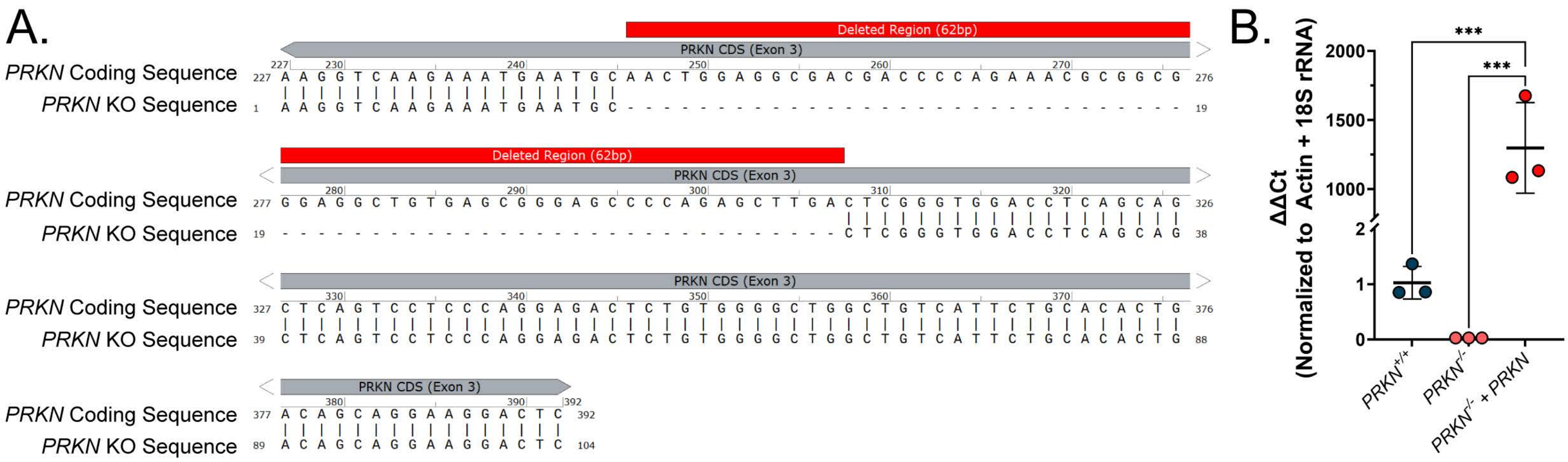

bioRxiv preprint doi: https://doi.org/10.1101/2024.05.21.594886; this version posted June 2, 2025. The copyright holder for this preprint (which was not certified by peer review) is the author/funder, who has granted bioRxiv a license to display the preprint in perpetuity. It is made available under aCC-BY 4.0 International license.

Figure S4

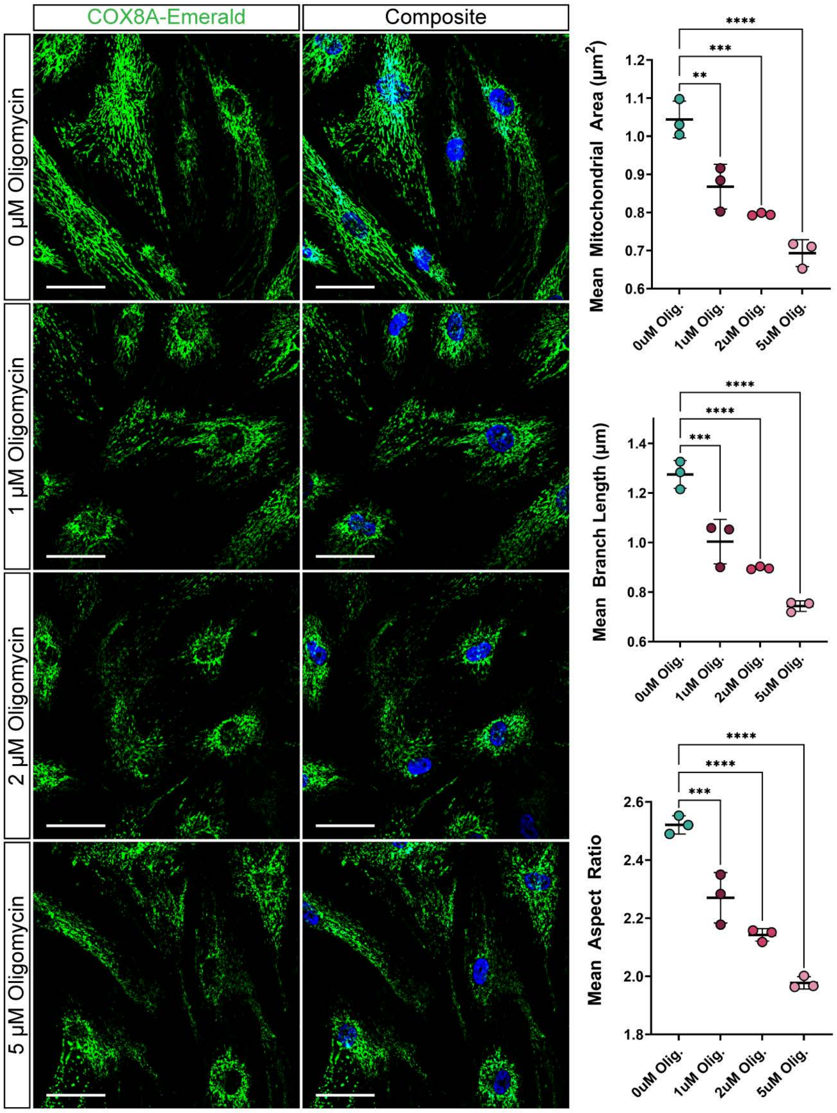

bioRxiv preprint doi: https://doi.org/10.1101/2024.05.21.594886; this version posted June 2, 2025. The copyright holder for this preprint (which was not certified by peer review) is the author/funder, who has granted bioRxiv a license to display the preprint in perpetuity. It is made available under aCC-BY 4.0 International license.

Plasmid Name	AddGene ID	Localization	Fluorophore	Antibiotic Resistance	Relevant Figures
pFUW H2B-mTagBFP2-p2A-PuroR	239552	Nucleus	mTagBFP2	Puromycin	1, 2, S1
pFUW H2B-Emerald-p2A-PuroR	239553	Nucleus	Emerald	Puromycin	2, S1, S2
pFUW H2B-mCherry-p2A-PuroR	239554	Nucleus	mCherry	Puromycin	2, S1
pFUW LAMP1Sig-Emerald	239555	Lysosome	Emerald	NA	1, 3
pFUW LAMP1Sig-mCherry	239556	Lysosome	mCherry	NA	1
pFUW COX8ASig-Emerald	239557	Mitochondria	Emerald	NA	1, 4, S4
pFUW COX8ASig-mCherry	239558	Mitochondria	mCherry	NA	1
pFUW PuroR-P2A-COX8ASig-Timer	239559	Mitochondria	Timer	Puromycin	5
pFUW COX8ASig-Lemon	239560	Mitochondria	Lemon	NA	S1
pFUW mito-GRX1-roGFP2	239561	Mitochondria	roGFP2	NA	6
pFUW mTagBFP2	239562	NA	mTagBFP2	NA	NA
pFUW Emerald	239563	NA	Emerald	NA	NA
pFUW mCherry	239564	NA	mCherry	NA	NA
pFUW mTagBFP2-P2A-PuroR	239565	NA	mTagBFP2	Puromycin	NA
pFUW Emerald-P2A-PuroR	239566	NA	Emerald	Puromycin	3
pFUW mCherry-P2A-PuroR	239567	NA	mCherry	Puromycin	NA
pFUW mTagBFP2-P2A-NeoR	239568	NA	mTagBFP2	Neomycin	NA
pFUW Emerald-P2A-NeoR	239569	NA	Emerald	Neomycin	NA
pFUW mCherry-P2A-NeoR	239570	NA	mCherry	Neomycin	NA
pFUW PRKN-P2A-PuroR	239571	NA	NA	Puromycin	6, S3

Table S1: A list of all plasmids generated in this study. Details for all plasmids, including subcellular localization, fluorophore, antibiotic resistance, and in which figure each plasmid was used. More plasmids were generated as part of GEM-SCOPe than used in the final publication, and these are also included here and available on Addgene.
